# Strengthening a Weak Tooth by Heavy Gold and Screws, Etc.

**Published:** 1879-12

**Authors:** George A. Mills

**Affiliations:** Brooklyn, N. Y.


### ARTICLE IV.

### *Strengthening Weak Teeth by Heavy Gold and Screws.—The use of Smooth Convex-Face Fillers.*

BY GEORGE A. MILLS, BROOKLYN, N. Y.

Read before the New Jersey Dental Society, at Long Branch, July 17, 1879.

From an experience and observation extending over a period of several years, I have become convinced that this matter has not received from our profession the attention to which its merits entitle it. I think the principal reason of this is, that *the real value of teeth* has not yet been fully appreciated.

A patient brought to the necessity of wearing an artificial denture has passed an unfortunate line, and one that cannot be recrossed. Just now, while I am writing, I am informed that one of New York City's most eloquent preachers—one who has electrified thousands in the church and lecture-room—is a sad example of this foreign invasion. I learn that the cause of his ruined pronunciation is a set of artificial teeth, and that he does not dare open his mouth freely for fear that they would drop from it; and they were inserted by one of New York's (so-called) best dentists.

To be sure, this case does not prove that all artificial dentures are failures; but it does prove that it is a great misfortune to be obliged to wear them. It was not necessary for him to have lost these teeth. They were lost because of the so-called "Rigg's disease," and, in such an organization as I personally know his to be, they were amenable to treatment.

Now to my subject. Let us take for example a lateral incisor—pulpless, decayed on both proximate sides, and involving most of the palatal surface. We will see at a glance that this is a very weak tooth. Now, to fill this according to the common method—replacing the portion destroyed by caries—would not add much to the strength of this weakened organ. Though such an operation is not infrequently made, yet too often it proves a failure, and the tooth is eventually lost.

I hold it to be a great failure of judgment to make such operations without the use of the strengthening auxiliaries which I have referred to in the heading of this article. The first step to be well considered is the preparation of the tooth for the operation, from the mechanical stand-point. After the health of the tooth has been provided for, we proceed to fill the root with cement; then, selecting such size and length of screw as are most suitable, enlarge the pulp canal to the depth required to retain it firmly; in many cases the length of it may be left undermined until near the completion of the operation. Platinum and iridium are the best materials to secure stiffness.

Having set the screw in the tooth (one as a rule will suffice for a lateral,) we so prepare it that direct access can be had and force applied in a line with the tooth. Then make grooves or slots at the base or the cervical part of the structure, in which to commence the operation we have in view. This step is by no means of the least importance, but is one of great nicety, in which thoroughness is demanded, for at this point strength must be secured. This is gained by the use of the screw, together with heavy gold.

Too many delude themselves with the idea of gaining strength with light forms of gold at the base. Is it reasonable to suppose that these, however nicely manipulated, can give the strength that Nos. 40, 60, or 120 rolled gold will secure? I do not think any mechanician will be in doubt as to which should be used.

We will now proceed to pack in these prepared places strips of 120 rolled gold (more often that than any lighter number.) Cut these pieces of gold nearly corresponding to the diameter of the place they are to occupy. Pack with a heavy lead mallet in the hand of a careful and trained assistant. I use a smooth convex-face filler, and am able literally to swage the gold into the form of slot described. Can any one conceive of such perfect adaptation, solidity, and strength so easily gained in any other way? On this base we begin our work, and proceed rapidly, observing extreme faithfulness in placing all portions of the material properly, thus restoring the lost form of the tooth,—either moulding the contour carefully as we progress, or else simply giving sufficient shape to it, so as to be able to form it afterwards in the finishing process. The use of convex fillers of proper shapes enables us to draw or strap the heavy gold firmly over the edges, securing the great advantage of the swaging principle, unequaled, I think, by any other.

If we have properly prepared this tooth, we have labial and palatal plates of enamel separated, and the screw extending down nearly or quite to the cutting edge. We have followed the proximate edges carefully, and arrived at a point where we must gain additional strength by clasping, so as to secure these plates from the possibility of being torn apart by the strain they will naturally be exposed to. This has been provided for by cutting off a line or more of the cutting edge of the tooth, which we now replace with gold. This completed, as it *can* be (or as Dr. S. G. Perry, of New York City, would say, "as it *should* be,") we are ready to finish with the help of disks, sand-paper, and the usual polishing materials, leaving a dead finish. This accomplished, we have but little exhibition of gold, and certainly a very substantial piece of work. As a rule, we have performed this operation (including the preparation) in about two hours, and not necessarily with a great amount of physical force expended by either the patient or operator.

I have selected a lateral incisor as an extremely weak tooth, coupled with the conditions named, viz., loss of structure and of the pulp. It must be apparent to any dentist of experience that to have confidence in such operations, as well as to fully appreciate the value of them after they are made, it must have been gained by a practice involving not a few defeats; but it is proved in many instances that the elements of success are found in these. I shall contend that such operations are of more value in such cases than any other, as a general rule, and that in utility, comfort, facial appearance, and durability they are superior to any artificial crown set upon roots. The effort should be to conserve all of the original structure compatible with the greatest amount of utility, and each operator will be governed by the standard of his own judgment and experience.

The auxiliaries of which I have spoken (screws and heavy gold) will find a very practical application to many other operations. It is quite common to find the bicuspids in a condition requiring them. If the pulp be lost, the loss of tooth-structure by caries extensive, and the cusps remain, either cut them off sufficiently or so prepare them that they may be safely secured. It has been said that heavy gold was necessarily a brutal thing to use. Such comments only give evidence of not understanding how to use it intelligently. I give the preference to rolled gold, knowing it to be better adapted to these uses; there is a tenuity to it that is not so perfect in the beaten gold.

It will be noticed that I have also given preference to my smooth convex-face fillers; this I do purposely, for by a constant use of them in general practice since June, 1870,

I have gained an experience that has brought to me *absolute knowledge* of their value. While they have been brought into quite extensive use, I am considerably surprised that a greater number do not perceive the mechanical principle involved more readily. Since introducing them to the profession, the same principle has been applied to the instrument used for caulking ships, and a royalty of 25 per cent. is paid to the inventor for all such tools manufactured for that purpose. Several dentists have applied the principle to fillers of their own especially favored forms. Dr. A. H. Brockway has a set of his own devising with the convex-face, as has also Dr. Knowles, of California.

I think it was in 1874 that a notice was published in the *Dental Cosmos*, signed by the chairman of the Committee on Operative Dentistry appointed by the American Dental Association, requesting information concerning improved methods, instruments, etc. I wrote out fully the history of these fillers, and described their use, principles, and value, forwarding it to the committee; but, to my surprise, when the report of the association appeared in print, not a word of reference to my contribution was embodied therein. Instead of that, a set of instruments was made mention of, designed by another dentist, with the same principle applied.

It has occurred to me that if I should use the term "gold caulkers" to these fillers the principle would be better understood by many, for in filling they are used literally for caulking gold into the cavities, slots, and crevices. As applied to heavy gold on the margins and beveled edges, they swage or strap it perfectly to the form of tooth-structure. Dr. Fletcher, of England, found in his efforts to produce water-tight fillings with cohesive gold, that he only succeeded by the use of the same principle (the round-faced filler, as he termed it, and so published it this year.) I have, for accommodation, had S. S. White apply the Varney serrations to my instruments, and find that the principle is still valuable to a considerable degree; but the fullest advantages are secured by keeping them smooth, for then the gold can be better adapted to the form of the tooth.

My purpose in putting these views before the profession is, that it may stimulate those who are ambitious to excel in that which costs more than the ordinary effort, and thus secure to our patients a greater amount of actual service, personal comfort, and satisfaction.—*Dental Cosmos.*

